# Modeling the natural history of fatty liver using lifestyle–related risk factors: Effects of body mass index (BMI) on the life–course of fatty liver

**DOI:** 10.1371/journal.pone.0223683

**Published:** 2019-10-21

**Authors:** Mika Aizawa, Seiichi Inagaki, Michiko Moriyama, Kenichiro Asano, Masayuki Kakehashi

**Affiliations:** 1 Department of Health Informatics, Graduate School of Biomedical & Health Sciences, Hiroshima University, Kasumi, Hiroshima, Japan; 2 International University of Health and Welfare, Narita, Chiba, Japan; 3 Department of Chronic Care and Family Nursing, Graduate School of Biomedical & Health Sciences, Hiroshima University, Kasumi, Hiroshima, Japan; 4 Human Resources Department Health Management Promotion Office, Fujikura Ltd. Kiba, Koto Ward, Tokyo, Japan; Beijing Key Laboratory of Diabetes Prevention and Research, CHINA

## Abstract

**Background:**

Incident fatty liver increases the risk of non**–**alcoholic fatty liver disease (NAFLD), which may lead to end-stage liver diseases, and increase the risk of cardiovascular disease and diabetes. For its prevention, modeling the natural history of fatty liver is useful to demonstrate which lifestyle-related risk factors (e.g. body mass index and cholesterol) play the greatest role in the life-course of fatty liver.

**Methods:**

Model predictors and their predictive algorithms were determined by prospective regression analyses using 5–year data from approximately 2000 Japanese men aged 20–69 years. The participants underwent health examinations and completed questionnaires on their lifestyle behaviors annually from 2012 to 2016. The life–course of fatty liver was simulated based on this participant data using Monte Carlo simulation methods. Sensitivity analyses were performed. The validity of the model was discussed.

**Results:**

The body mass index (BMI) and low–density/high–density lipoprotein cholesterol (LDL–C/HDL–C) ratio significantly aided in predicting incident fatty liver. When the natural history of fatty liver was simulated using the data of participants aged 30–39 years, the prevalence increased from 20% to 32% at 40–59 years before decreasing to 24% at 70–79 years. When annual updates of BMI and LDL–C/HDL–C ratio decreased/increased by 1%, the peak prevalence of fatty liver (32%) changed by −8.0/10.7% and −1.6/1.4%, respectively.

**Conclusions:**

We modeled the natural history of fatty liver for adult Japanese men. The model includes BMI and LDL‒C/HDL‒C ratio, which played a significant role in predicting the presence of fatty liver. Specifically, annual changes in BMI of individuals more strongly affected the life‒course of fatty liver than those in the LDL–C/HDL–C ratio. Sustainable BMI control for individuals may be the most effective option for preventing fatty liver in a population.

## Introduction

Fatty liver is an adaptation to lipid loading in the liver, which increases the risk of non–alcoholic fatty liver disease (NAFLD). It is currently the most common form of chronic liver disease [1]. In recent decades, NAFLD has become widespread, with a steady increase correlating with the rise in obesity worldwide [2]. The prevalence of NAFLD is estimated at 27%–34% in North America, 25% in Europe and 15%–20% in Asia [3]. In Japan, NAFLD is reported to affect 13%–30% of the general population [4]. NAFLD may slowly progress to end–stage liver diseases such as liver cirrhosis and hepatocellular carcinoma [5]. In addition, NAFLD is an independent risk factor for cardiovascular disease and type 2 diabetes [6–9]. Its potential to progress to such diseases makes the prevention of fatty liver important.

To prevent fatty liver, lifestyle modification is highly recommended. Several lifestyle-related factors are associated with incident fatty liver such as alcohol intake [10], lower physical activity [11], high body mass index (BMI) [12], low high–density lipoprotein cholesterol (HDL–C) [13], high triglycerides [13,14], high low–density lipoprotein cholesterol (LDL–C) [15], type 2 diabetes mellitus [16], smoking [17] and shift–work [18]. Furthermore, previous prospective studies revealed several important findings. Lallukka et al. reported that baseline liver fat and changes in BMI were independent predictors of liver fat. [19]. Zelber–Sagi et al. reported that changes in weight are a strong predictor for NAFLD development/remission [20]. However, the effects of such preventable risks through lifestyle modification on the life–course of fatty liver are unclear.

To investigate their effectiveness, a simulation approach is considered useful. Several modeling studies have been used for disease prevention and management [21–24]. For example, Estes et al. evaluated the NAFLD disease burden to model its progression [25], and Vreman et al. estimated the health and economic benefits of reducing sugar intake using a model for the progression of NAFLD [26]. However, a simulation model of incident fatty liver has not been developed using lifestyle–related risk factors.

This study aimed to model the natural history of incident fatty liver using lifestyle–related risk factors for Japanese men and investigated which factors most strongly affected the life–course. In this study, we focused on Japanese men because gender is known to have significant effects on the prevalence of NAFLD. For example, NAFLD was most prevalent for men in their 40s, whereas for women it was most prevalent in those over 50 years of age [27]. In addition, the average prevalence of NAFLD was observed to be two–times [4] and three–times [28] higher among men than among women.

As nationwide examination for fatty liver is not practiced in Japan, few observational datasets of fatty liver prevalence were reported [4, 28, 29]. For our study, we obtained 5–year data from a study population consisting of approximately 2000 Japanese adult men for modeling, and to assess the internal validity and the sensitivity of the model. We report (1) the representativeness of the study population for adult Japanese men, (2) model development, (3) simulation and model assessment, and (4) the sensitivity analysis. Additionally, we discuss the validity of our model in detail.

## Data and methods

### Study population

The study population consisted of Japanese men aged 20–69 years who participated in health examinations and completed questionnaires on their lifestyle behaviors annually from 2012 to 2016 (n = 1891, 1949, 2036, 2063 and 2240 for the years 2012‒2016, respectively). The entire population was composed of employees from Fujikura Ltd., a large manufacturer in the Japanese power and telecommunications industry. Recruitment of the study participants was almost closed through the observation years; approximately 9% of the participants annually entered the study, whereas 6% of the participants annually left the study, due to retirement/employment, career change or the absence of annual health examinations. We excluded 4.6% (≥40 years) and 17.4% (<40 years) of the population from the analysis due to missing clinical, biochemical and lifestyle data. The treatment of missing fatty liver data is detailed in the next section.

### Variables used for analyses

The data collected from the study population included the following variables: health status (age, diagnosis of fatty liver, BMI, LDL–C, HDL–C, triglycerides (TG), systolic blood pressure (SBP) and hemoglobin A1c (HbA1c)) and lifestyle (smoking, alcohol drinking, exercise and shift work). The data on health status were obtained through annual health examinations. Those on lifestyle were obtained from self–report questionnaires distributed during the health examination (see S1 File).

The definitions of the variables are as follows (diagnosis of fatty liver, BMI, LDL–C, HDL–C, TG, SBP and HbA1c were based on commonly used clinical criteria. The definitions of smokers and exercisers were based on those used in the specific health examinations in Japan. The definition of alcohol drinkers followed the clinical practice guidelines for nonalcoholic fatty liver disease/nonalcoholic steatohepatitis [30]):

1. The diagnosis of fatty liver was made by physicians based on ultrasonography [30].
2. Clinical and biochemical data (BMI, LDL–C, HDL–C, TG, SBP and HbA1c) were measured by standard procedures used during general health examinations.
3. Smokers were defined as people who had been smoking for ≥6 months or consuming ≥100 cigarettes for at least 1 month.
4. Alcohol drinkers referred to men whose average daily alcohol consumption was ≥30 g/day. We categorized someone as an alcohol drinker when their daily alcohol consumption (defined as gross consumption of Japanese sake) and drinking frequency were “>360 ml” and “3–4 days a week” or “≥180 ml” and “5–7 days a week”.
5. Exercisers referred to people who exercised with moderate intensity for ≥30 min continuously at least 2 times a week for one year.
6. Shift workers are people engaged in manufacturing–related blue–collar work, including night shifts.

The availability of fatty liver data for participants was 0% for those aged 20–29 years and 9.1% for those aged 30–39 years. In contrast, 95.5% of the study population aged 40–69 years had available fatty liver data. Missing fatty liver data of those who were 30–39 years (n = 1981) were estimated by applying multivariate logistic regression analysis using the data collected from the participants in the age range of 30–69 years. The estimation of missing data and the validity are shown in S2 File.

### Determination of model structure

The predictive algorithms for both the presence or absence of fatty liver and its predictor variables were determined by multivariate regression analyses between *V_i_*(t) and *V_j_*(t+1) using the 5–year data of the study population (for the details, see S4 File). The variables (*V*) we used for analysis were: the presence of fatty liver, age, BMI, LDL–C, HDL–C, LDL–C / HDL–C, TG, SBP, HbA1c, smoking, alcohol drinking, regular exercise and shift work. To predict the presence of fatty liver in the regression, continuous variables (BMI, LDL–C, HDL–C, LDL–C / HDL–C, TG, SBP and HbA1c) were categorized as normal/abnormal based on guidelines or references commonly used in risk stratification (see S3 File).

### Simulation and model assessment

Model performance is often supported by evidence gathered from comparing projected outputs to internal data (used to develop the model) and to external data (not used to develop the model) [31]. In this study, we performed the following assessments: (V–1) model calibration, (V–2) internal consistency of the simulated natural history of fatty liver, and (V–3) external consistency of the simulated natural history model of fatty liver. For these assessments, we established four types of theoretical cohorts, 1‒4, by selecting participants from the study population as described below.

To calibrate the model (V–1), we established cohort 1, which excluded all individuals in the study population who had missing data throughout the observation years from 2012 to 2016 (n = 982). The values of predictors and the presence of fatty liver for each member of cohort 1 were annually predicted from 2012 to 2016. The simulated prevalence as trends of predictors and incident fatty liver was compared with the observed prevalence in the cohort itself. When the observed prevalence fell within the Monte Carlo variation of the mean simulated prevalence, we considered the simulated prevalence consistent with the observed prevalence.

For (V–2), time‒shift simulations were performed because the prevalence of fatty liver changes with age. We established theoretical cohorts 2, 3 and 4, which consisted of individuals who were 30‒39 years (n = 2162), 40‒49 years (n = 3650) and 50‒59 years (n = 1845), respectively, in the observation period from 2012 to 2016. The life‒course of fatty liver was simulated for cohorts 2, 3 and 4 until the cohort members reached 70‒79 years of age, given that the mean life expectancy was approximately 40 years for the Japanese men who were 39 years of age in 2012 [32]. Each projected prevalence in cohorts 2–4 was compared with the observed prevalence. In contrast to cohort 1, the data for comparison were not observed in the cohort itself but in the study population of the same age groups (30–39, 40–49, 50–59 and 60–69 years).

Regarding (V–3), the life–course of fatty liver simulated for cohort 2 was compared with the fatty liver prevalence in different age groups from 3 types of external data observed in men who: received a health check–up in Okinawa prefecture in Japan in 1984 [29], visited the Tokai University Hospital Health Checkup Center in Japan from 1989 to 2000 [4], or received a health check–up at health centers in Japan from 2009 to 2010 [28].

Sensitivity analysis was performed by varying the following aspects: (S–1) the initial characteristics of cohort 2 and (S–2) predictive algorithms of the predictor variables for fatty liver. Regarding (S–1), we examined the effects of variations (±20% and ±40%) in the initial characteristics of cohort 2: the prevalence of subjects with a BMI ≥25 kg/m^2^, LDL–C ≥120 mg/dl, HDL–C <40 mg/dl, LDL–C/HDL–C ≥2, TG ≥150 mg/dl, SBP ≥130 mmHg, HbA1c ≥6.5%, smoking, alcohol drinking, regular exercise and shift work. Theoretical cohorts with these varied characteristics were established by sufficient sampling from the study population.

Regarding (S–2), we examined the effects of variation in predictive algorithms of the predictor variables on the annual prediction of fatty liver. For example, the predictive algorithms were changed to provide a 0.5 and 1% increase/decrease from the annual updates to investigate which predictor variables most strongly affected the life–course of fatty liver.

### Simulation software

The software “Mathematica 11.03” was used to perform all the simulations. The projections were performed using the Monte Carlo simulation method. The projected prevalence was obtained as the mean prevalence calculated through 10–time runs of the model for individuals at one–year intervals. The standard deviation (SD) of Monte Carlo variation (2 SD) was also calculated.

### Statistical analysis

The software “EZR 1.36 for Windows” was used to perform all statistical analyses. Multivariate logistic or linear regressions were conducted by forced entry and the backward elimination method. The quantitative data were expressed as mean values ±SD. The Student’s t–test was used to perform the comparisons between groups of quantitative variables. The Chi–squared test was used to perform contingency table analyses. A two–tailed p-value p<0.05 was considered significant.

## Results

### Construction of the model

#### The characteristics of the study population for model construction and assessment

The study population (approximately 2000) was much smaller than the entire population of Japanese men, which may have caused selection bias. Therefore, the characteristics of the study population were investigated to assess whether the population represented general Japanese men. The study population was compared with a survey population (See Table 1). The clinical and biochemical characteristics of the study population were almost comparable to those of general Japanese men. Therefore, we concluded that the study population was representative of general Japanese men.

**Table 1:** Clinical and biochemical characteristics of the study population and a national survey population.

	The Study Population		Survey population [35]	
	Mean	SD	Mean	SD
BMI (kg/m^2^)	23.6	3.5	23.6	3.7
LDL–C (mg/dl)	122.2	29.8	121.7	29.5
HDL–C (mg/dl)	56.9	13.4	55.4	14.3
HbA1c (%)	5.4	0.5	5.5	0.5
TG (mg/dl)	120.3	86.6	165.1	122.4
SBP (mmHg)	122.3	14.7	126.3	14.0

The study population consisted of men from 20–69 years of age (41.8 ± 9.9 years), n = 10179 person·years (from 2012 to 2016). For the survey population, all data were adjusted to match the age distribution of the study population.

### Model variables and their predictive algorithms

For modeling, variables and their predictive algorithms were determined by conducting multivariate regression analyses. The lifestyle-related variables that strongly aided in the annual prediction of fatty liver were BMI and LDL–C/HDL–C ratio. The predictive algorithms of the presence of fatty liver, BMI and LDL–C/HDL–C ratio are detailed in S4 File.

### Transition probabilities to project the fatty liver prevalence

In this study, microsimulations were performed to project the fatty liver prevalence. For the simulation, the transition probabilities for the presence of fatty liver were calculated (See Table 2). In the use of the predictive algorithms mentioned above, the probabilities were calculated using non–standardized regression coefficients of predictor variables and a constant term, which were both provided through the multivariate logistic regression (See S4 File). The transition probabilities were 0.072 (if BMI≥25 kg/m^2^) and 0.074 (if LDL/HDL ≥2). If individuals had a BMI≥25 kg/m^2^ and LDL/HDL≥2, the probability increased to 0.164. All calculations were based on main effects. Interaction terms of the predictor variables were not significant. Although fatty liver was present before transition, the transition probabilities were less than 1.0. This suggests that our model can reproduce the clinical evidence that fatty liver is reversible between incidence and remission [30]. All the projections of fatty liver prevalence were carried out using the transition probabilities to predict the presence of fatty liver individually.

**Table 2:** Transition probabilities for the presence of fatty liver determined by the predictor variables before transition.

Predictor variables (*) before transition			Estimated transition probabilities of fatty liver presence
Fatty liver	BMI≥25 (kg/m^2^)	LDL–C/HDL–C≥2	
0	0	0	0.031
0	0	1	0.074
0	1	0	0.072
0	1	1	0.164
1	0	0	0.649
1	0	1	0.823
1	1	0	0.819
1	1	1	0.919

(*) Predictor variable = 0 (if positive), 1 (if negative)

### Simulation and model assessment

#### The characteristics of cohorts 1–4 for projection and assessment

Cohorts 1–4 were theoretically created by selecting participants from the study population for the purposes of projecting fatty liver prevalence and assessment of the model. The characteristics of cohorts 1–4 are presented in Table 3.

**Table 3:** The initial characteristics of cohorts 1‒4.

	Cohort 1		Cohort 2		Cohort 3		Cohort 4	
n (person)	982		2162		3650		1845	
	Frequency (%)							
Age (year)								
30–39	3.0		100.0		0.0		0.0	
40–49	65.7		0.0		100.0		0.0	
50–59	29.3		0.0		0.0		100.0	
60–69	2.0		0.0		0.0		0.0	
Total	100.0		100.0		100.0		100.0	
	Prevalence (%)							
Fatty liver	27.9		19.8		30.5		29.1	
Smoking	32.0		35.4		32.9		31.3	
Alcohol drinking	39.2		28.4		35.9		48.1	
Regular exercise	21.1		18.6		21.4		26.1	
Shift work	33.3		41.4		43.2		26.3	
	Mean	SD	Mean	SD	Mean	SD	Mean	SD
Age (year)	47.3	5.6	35.4	3.0	44.7	2.8	54.2	2.8
BMI (kg/m^2^)	24.0	3.3	23.3	3.6	24.0	3.4	24.0	3.3
LDL-C (mg/dl)	125.3	28.4	118.5	29.5	126.9	29.6	125.2	28.6
HDL-C (mg/dl)	55.7	13.4	56.6	13.3	56.0	14.3	57.5	13.9
TG (mg/dl)	135.2	101.4	111.3	82.9	127.4	91.5	132.0	101.3
HbA1c (%)	5.4	0.6	5.2	0.4	5.4	0.6	5.5	0.6
SBP (mmHg)	124.0	16.7	121.4	14.1	123.6	16.1	126.3	17.0

**Fig 1:**
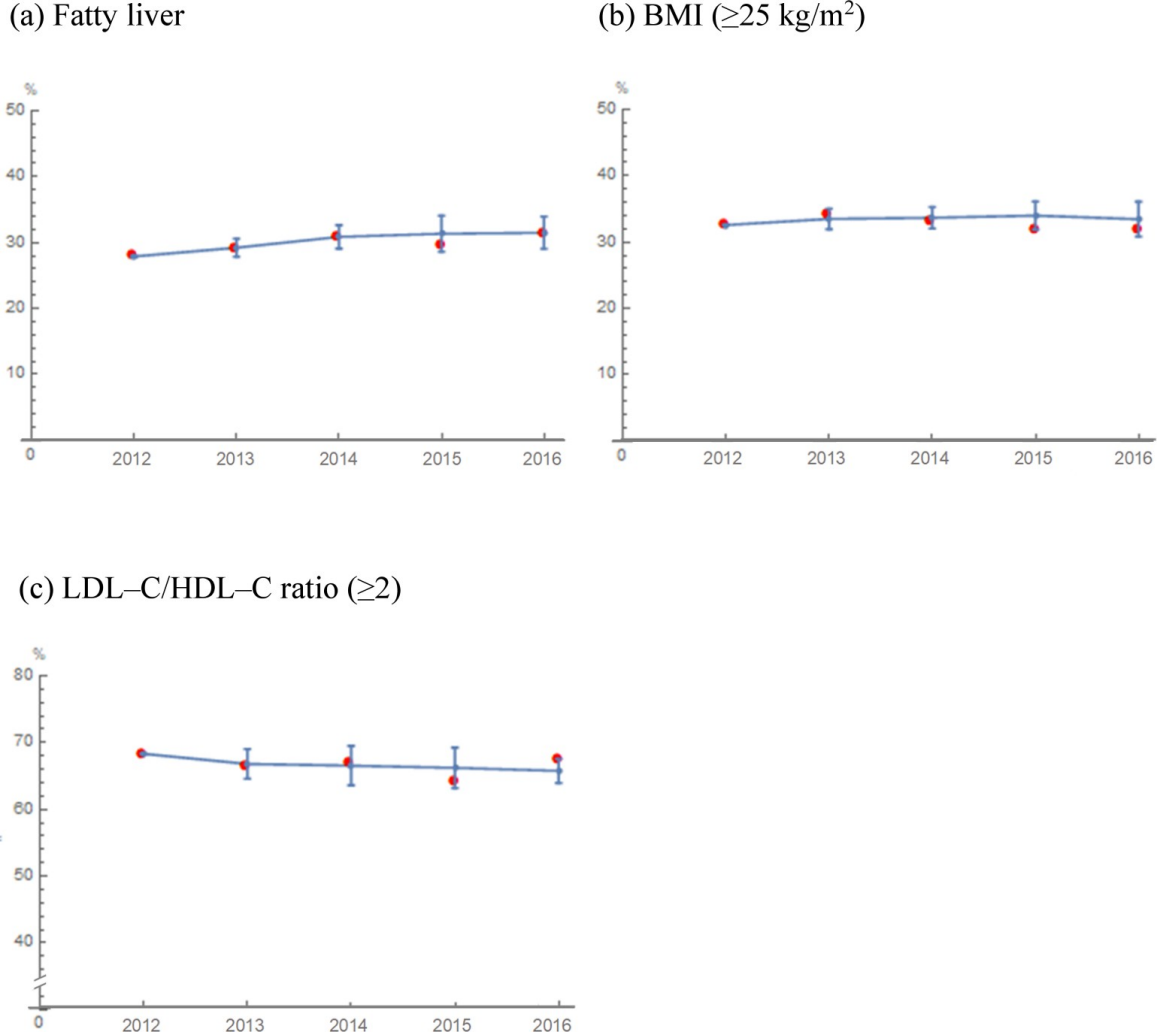
**Projected prevalence in cohort 1 from 2012 to 2016: (a) Fatty liver, (b) BMI (≥25kg/m^2^) and (c) LDL–C/HDL–C ratio (≥2).** The trajectories of the projected prevalence (blue curve) are illustrated. Error bars indicate the mean prevalence within ±2 SD as Monte Carlo variations. Red dots indicate the mean prevalence in the study cohort observed in 2012‒2016.

For cohort 1, the projected prevalence of fatty liver, BMI (≥25 kg/m^2^) and LDL–C/HDL–C ratio (≥ 2) are shown in Fig 1. Each observed prevalence in cohort 1 fell within ±2 SD of the predicted mean prevalence of fatty liver, BMI (≥25 kg/m^2^) and LDL–C/HDL–C ratio (≥ 2). This suggested that our model reproduced the observed prevalence of fatty liver by suitably predicting the BMI and cholesterol ratio according to their predictive algorithms.

**Fig 2:**
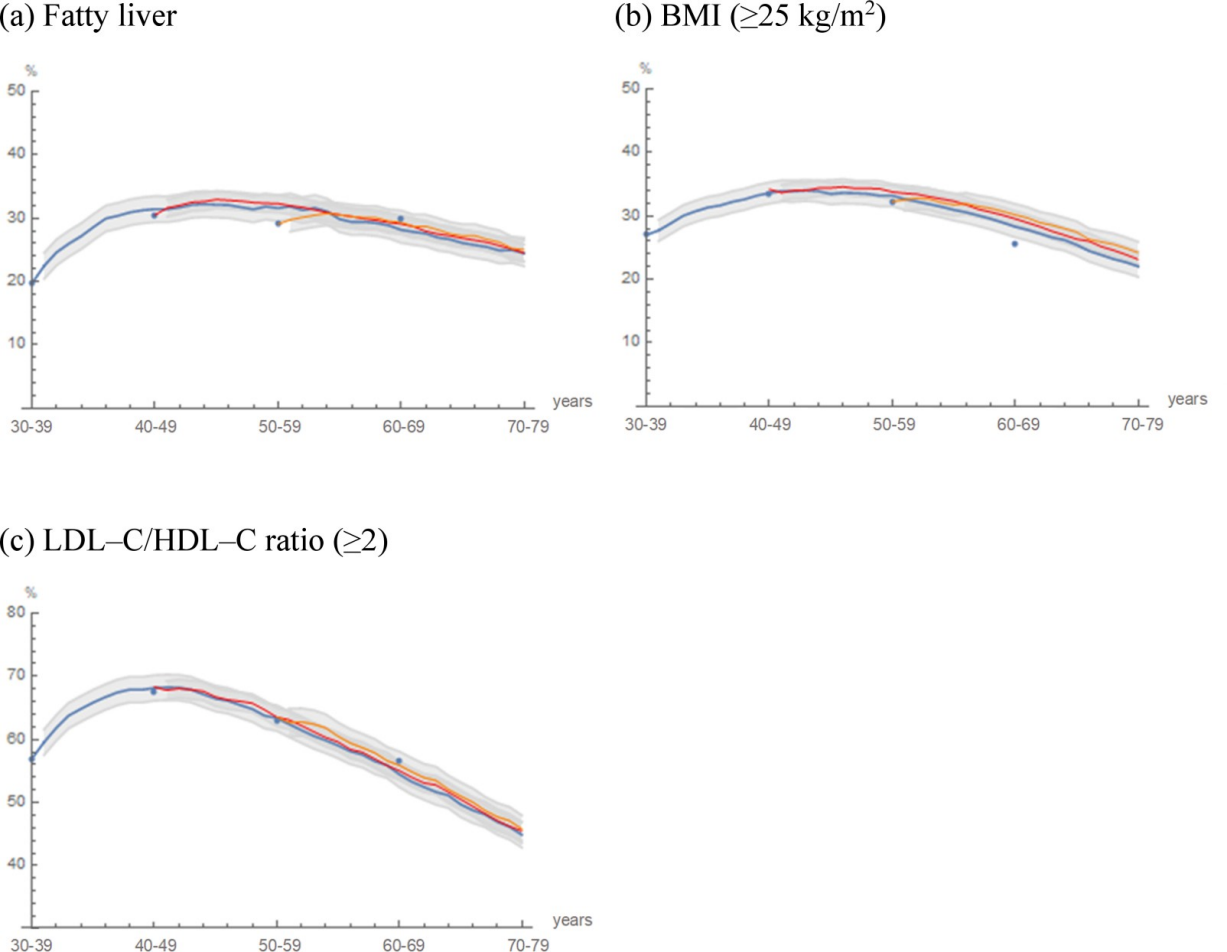
**Projected prevalence in cohorts 2–4: (a) Fatty liver, (b) BMI (≥25 kg/m^2^) and (c) LDL–C/HDL–C ratio (≥2).** The trajectories of the projected prevalence in cohort 2 (blue curve), in cohort 3 (red curve) and in cohort 4 (Orange curve) are illustrated. Areas in gray indicate the mean prevalence within ±2 SD as Monte Carlo variations. Blue dots indicate the mean prevalence in the study population observed in 2012‒2016 (n = 10179).

For modeling the natural history of fatty liver, further projection and assessment were performed. For cohorts 2–4, the projected prevalence of fatty liver, BMI (≥25 kg/m^2^) and LDL–C/HDL–C ratio (≥ 2) are shown in Fig 2. Each observed prevalence in the study population of the same age group fell almost within ±2 SD of the projected mean prevalence in cohort 2. Furthermore, the trajectories of prevalence in cohort 2 almost overlapped with those in cohorts 3 and 4. As illustrated in the Fig 2, the cohort effects were negligible between cohorts 2–4 and the study population. In addition, the fatty liver data of cohort 3 included estimated fatty liver data. However, there was no significant difference observed in the projected prevalence among cohorts 2, 3 and 4. Therefore, projection using estimated fatty liver data of cohort 2 was concluded to be highly reliable.

**Fig 3:**
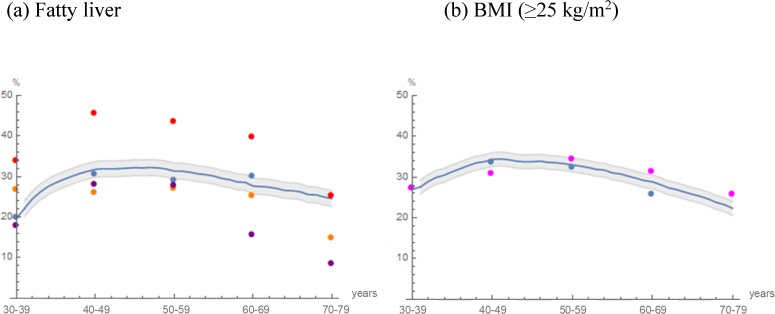
**Projected prevalence in cohort 2 and recorded prevalence in external cohorts: (a) Fatty liver, (b) BMI (≥25 kg/m^2^).** The trajectories of the projected prevalence in cohort 2 (blue curve) are illustrated. Areas in gray indicate the mean prevalence within ±2 SD as Monte Carlo variations. Blue dots indicate the prevalence in the study population recorded in 2012‒2016 (n = 10179). Observed external cohort data were indicated by purple [29], orange [4], red [28] and magenta dots [35].

For further confirmation of model validity, the projected prevalence of fatty liver and BMI were compared with those of external cohorts. As shown in Fig 3, the observed prevalence in each age class varied widely depending on the observed period and population. We are unsure why the reported prevalence of fatty liver in Japanese men varied from 12.6% to 41.0% [4,28, 29]. The following may be reasons: referral bias, modality for diagnosis, observed year and population heterogeneity [33, 34].

The projected prevalence of fatty liver increased from 19.8% and peaked at 31.5% before decreasing to 24.9%, which was in the range of the reported prevalence in the external cohorts. At 70–79 years of age, the prevalence of fatty liver in external cohorts markedly decreased, whereas the projected prevalence of fatty liver only slightly decreased. In contrast, the predicted prevalence of a BMI ≥25 kg/m^2^ fit better with the observed data from the survey population and the study population, as shown in Fig 3. The projected prevalence of fatty liver was comparable with that observed in the external cohort whose data were not used to construct the fatty liver model. This finding may further increase the reliability of the projected fatty liver prevalence.

### Sensitivity analysis

**Fig 4:**
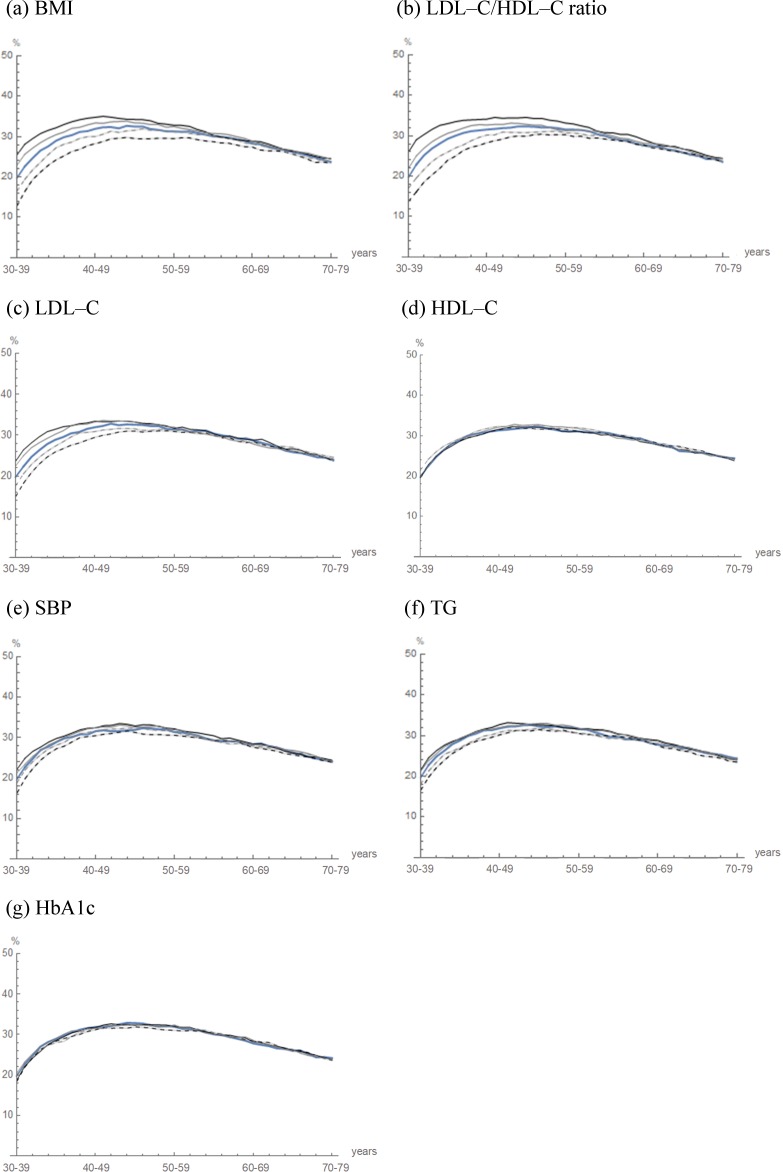
Simulated life–course of fatty liver with variations by changes in the clinical and biochemical predictor variables of cohort 2. The trajectories of the projected prevalence in cohort 2 (blue curve) are illustrated. The initial proportion of the clinical and biochemical predictor variables of cohort 2 were varied by −40% (dashed black curve), −20% (dashed gray curve), 20% (gray curve) and 40% (black curve).

**Fig 5:**
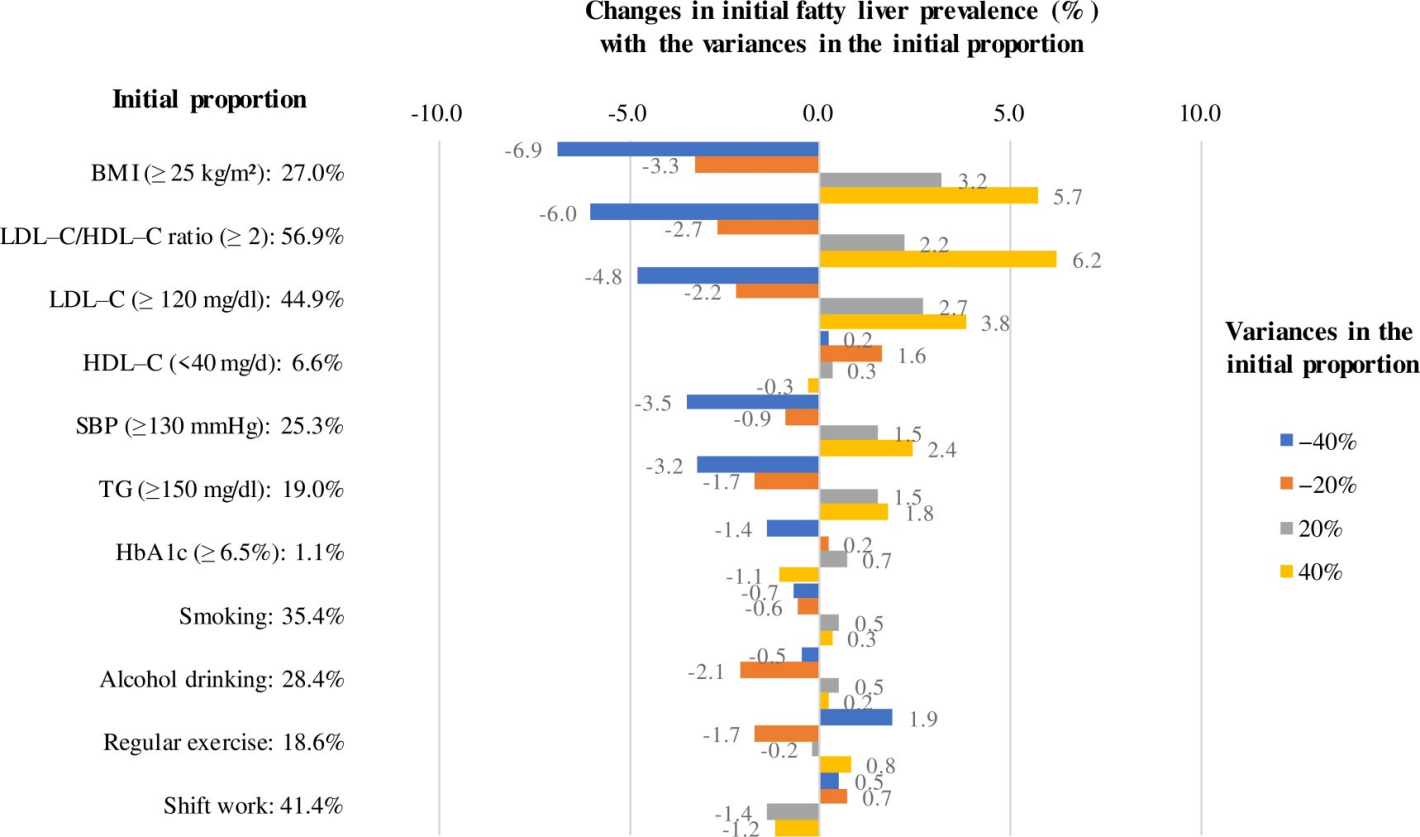
Changes in fatty liver prevalence with varying initial proportions of the predictor variables of cohort 2.

After validity was confirmed, sensitivity analysis was performed by varying the following 2 aspects: the initial characteristics of cohort 2, and the predictive algorithms of BMI and LDL–C/HDL–C ratio. First, when the initial characteristics of cohort 2 were varied by ±20% and ±40%, the prevalence of fatty liver changed, as illustrated in Figs 4 and 5. The variation in the prevalence of fatty liver was largest before projection, and steadily decreased as projection years increased. The maximum amount of change in the fatty liver prevalence caused by such variations is shown in Fig 5. In particular, the BMI and LDL–C/HDL–C ratio strongly affected the life–course of fatty liver. For example, when the proportion of those with a BMI ≥25 kg/m^2^ was varied from 27.0% to 16.2%/37.8% (40% decrease/increase), the prevalence of fatty liver changed by −6.9%/5.7%. When the proportion of those with an LDL–C/HDL–C ratio ≥2 was varied from 56.9% to 34.1%/79.7% (40% decrease/increase), the prevalence of fatty liver changed by −6.0%/6.2%. In contrast, the following variables HDL–C, HbA1c, alcohol drinking, smoking, regular exercise and shift work only altered the fatty liver prevalence by less than 2.0%, which was almost within 2 SD of the Monte Carlo variation of the projected mean fatty liver prevalence in the projection period (see Fig 5). The changes in the initial characteristic of a population, especially the BMI and LDL–C/HDL–C ratio, greatly affected the life–course of fatty liver. However, their effectiveness decreased as projection continued into the future.

**Fig 6:**
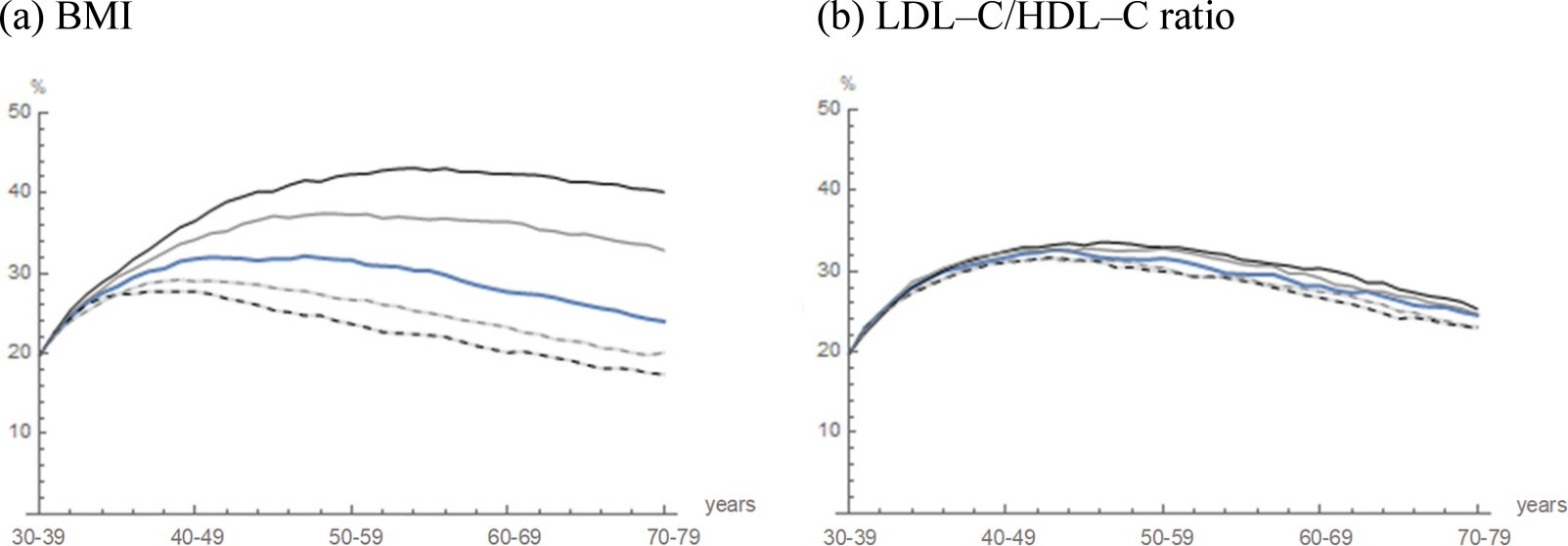
Simulated life–course of fatty liver with variations by changes in the predictive algorithms of BMI and LDL–C/HDL–C ratio. The trajectories of the projected prevalence in cohort 2 (blue curve) are illustrated. The mean prevalence in which the predicted BMI and LDL–C/HDL–C ratio were varied by −1.0% (a dashed black curve), −0.5% (a dashed gray curve), 0.5% (a solid gray curve) and 1.0% (a solid black curve).

In the second step, we examined the effects of variations in annual updates of BMI and LDL–C/HDL–C ratio on the life–course of fatty liver. When annual updates of BMI and LDL–C/HDL–C ratio decreased/increased by 0.5% and 1.0%, the life–course of fatty liver changed, as shown in Fig 6. The impact of the changes in annual updates of BMI and LDL–C/HDL–C ratio became larger as the projection continued into the future, whereas the impact of the changes in initial characteristics of cohort 3 became smaller. In addition, annual changes in BMI affected the life–course of fatty liver greater than those in the LDL/HDL–C ratio. For example, if annual updates of BMI decreased/increased by 1.0%, the prevalence of fatty liver changed by −8.0%/10.7%. In contrast, if annual updates of the LDL–C/HDL–C ratio decreased/increased by 1.0%, the prevalence of fatty liver changed by −1.6%/1.4% (see Table 4).

**Table 4:** Changes in fatty liver prevalence with variances in the predicted BMI and LDL–C/HDL–C ratio.

	Changes in the fatty liver prevalence (%) (*)			
Variation	−1.0%	−0.5%	+0.5%	+1.0%
BMI (kg/m^2^)	−8.0	−5.0	5.6	10.7
LDL‒C/HDL‒C ratio	−1.6	−1.2	1.2	1.4

(*) Changes in the fatty liver prevalence with variations (±0.5% and ±1.0%) in BMI and LDL–C/HDL–C ratio for cohort 2 at the age of 50–59 years old.

## Discussion

To our knowledge, this is the first study to model the natural history of fatty liver using lifestyle-related risk factors. The model demonstrated the life‒course of fatty liver as a function of the presence of fatty liver, BMI and LDL‒C/HDL‒C ratio. Sensitivity analysis illustrated the importance of BMI as a risk factor for fatty liver, with annual changes in BMI more strongly affecting the life–course of fatty liver than those in the cholesterol ratio.

In general, disease modeling enables us to estimate the long-term disease prevalence and the effectiveness of different interventions on disease prevention in a time- and cost-saving manner. However, a common weakness associated with such models is that implicit/explicit assumptions are inherent (e.g., the particular values for parameters and the underlying/causal relationships for the model structure). Therefore, model validation is important. Kopec et al. suggested a framework for model assessment after reviewing the concepts and methods for validation of population‒based disease simulation models [31]. In accordance with this framework, the validity of our model was confirmed by gathering evidence on the following aspects: underlying theories/assumptions, definition of variables, model content, structure and parameters (to examine the model development process), between‒model comparisons, parameter sensitivity, internal consistency and comparisons with external data (to examine model performance). In many modeling studies, the underlying relationships between input values and outcomes of the model were treated as a “black box”. To address this critique, we took a transparent approach by systematically investigating the underlying relationships based on recorded data, and determined the model structure and parameters (we reported the results in S4 File). In the following sections, we discuss our model validity in detail.

### Assessment of the model developing process

#### Underlying theories

For underlying theories, our model incorporated several concepts from medicine and epidemiology. For example, baseline liver fat and changes in BMI are independent predictors of liver fat [19] and changes in weight are a strong predictor for NAFLD development/remission [20]. In addition, our model incorporated concepts of Monte‒Carlo simulations. These concepts are based on accepted phenomena of fatty liver and a widely accepted theory of stochastic simulations.

#### Underlying assumptions

Underlying assumptions for model development were related to the following characteristics: (1) population-based model (ours was a microsimulation model for Japanese men), (2) updated risk factors (risk factors were annually updated using their predictive algorithms), (3) multiple risk factors (the co‒occurring risk factors contributed independently to estimate the presence of fatty liver) and (4) closed population (projection cohort members were all alive during the simulation period). Assumptions (1) ‒ (3) were acceptable for our study aim and scope. Microsimulation approaches and annual updated multiple risk factors are commonly used in population‒based disease modeling [21–26]. In the case of (4), a closed population (the cohort members are all alive during the simulation period), the simulated disease outcomes may be slightly overestimated partly due to deaths not being incorporated. Therefore, this assumption may be a limitation that compromises the model’s credibility. We explain the shortcomings in more detail in the limitations section.

#### Definition of the variables

The diagnosis of fatty liver, BMI and LDL-C/HDL-C ratio were based on commonly used clinical criteria. The BMI and LDL‒C/HDL‒C ratio were categorized as normal/abnormal based on guidelines or references commonly used in risk stratification (see S1 and S3 Files).

#### Model content and structure

The variables included in the model were the presence of fatty liver, BMI and LDL‒C/HDL‒C ratio based on the regression analysis (see S4 File). For example, the presence of fatty liver at t (year) was a predictor for incident fatty liver at t+1 (year). This is consistent with accepted clinical phenomena that baseline liver fat and changes in BMI are independent predictors of liver fat [19]. In addition to such evidence, a prospective cohort study revealed that BMI was a factor for the onset and remission of NAFLD [20]. Our study substantiates these findings as it demonstrated that BMI is a valid predictor of fatty liver. In contrast, there was no previous evidence of the association of LDL–C/HDL–C ratio with fatty liver. To our knowledge, ours is the first study to report that the LDL–C/HDL–C ratio played a stronger role in the annual prediction of fatty liver than LDL–C and HDL–C alone (see the S4 File). Although our finding is not yet well established, we believe that some previously observed phenomena support the validity of the LDL–C/HDL–C ratio as a predictor of fatty liver. Sun et al. found that a high LDL–C was associated [15] with incident fatty liver (higher HDL–C was inversely associated [13]). In addition, Manninen et al. [36] and Enomoto et al. [37] reported that the LDL–C/HDL–C ratio was strongly correlated with the onset of coronary heart disease (CHD) and atherosclerosis progression. A meta–analysis confirmed an independent association between NAFLD and increased CHD prevalence [38]. Recently, new types of lipid parameters, triglyceride to HDL–C ratio [39] and non–HDL–C to HDL–C ratio [40], were reported to be strong predictors of incident fatty liver. Further studies will clarify the association of the cholesterol ratio with the increased risk of incident fatty liver.

Contrary to the aforementioned variables, HbA1c, regular exercise, smoking, alcohol drinking and shift-work were excluded from our model based on our regression analysis (See S4 File). For example, although an HbA1c ≥6.8% was highly prevalent in patients with NAFLD [7], it was not significant in the annual prediction of fatty liver. The prevalence of fatty liver peaks in those in their 40s‒50s; however, the number of cases of higher HbA1c (≥6.8%) gradually increase with age, as confirmed by Japanese national health statistics [35]. Therefore, it is unlikely that HbA1c has a large role in the annual prediction of fatty liver. In contrast to several previous studies reporting beneficial effects of exercise on liver fat [11,41], we excluded exercise from our model. This was partly because of the lower dose (intensity, frequency and volume) of exercise we categorized in this study. In addition, exercise for study participants was not controlled and only reflected individual lifestyle behaviors, which were self–reported. Lastly, alcohol drinking, smoking, and shift–work were also not incorporated in the model. These factors were associated with fatty liver [10, 17, 18]; however, strong evidence for these factors as predictors of fatty liver has not been reported. Conclusively, after excluding the above variables, the internal validity and consistency of the projected fatty liver prevalence was confirmed using external data.

### Assessment of model performance

#### Between–model comparisons and parameter sensitivity

After discussing the validity of the model development process, we evaluated our model performance by between–model comparisons and in terms of parameter sensitivity. Our model included BMI and LDL–C/HDL–C ratio as predictors of fatty liver trends. First, we compared our BMI trends with those from a Canadian microsimulation model, the Population Health Model for Cardiovascular Disease [42]. This model captured the life‒course of BMI, demonstrating an increase in BMI up to middle-age, followed by a slow decrease in older age. The trends we simulated were consistent with this Canadian model. Furthermore, our BMI estimates were comparable with external records from a Japanese survey, the National Health and Nutrition Examination Survey [35]. Second, the annual updates of the LDL–C/HDL–C ratio were performed in the same manner as those of BMI (The simulations were performed stochastically using predictive algorithms derived from regression analysis using recorded data). This approach is common in risk factor/disease prediction performed using other models, [43–46]. The trends of the LDL–C/HDL–C ratio were unable to be compared with those from other models due to their unavailability. However, model credibility was assessed in terms of cholesterol sensitivity. The sensitivity of the cholesterol ratio to the life‒course of fatty liver was much lower than that of BMI. For example, a 1% decrease/increase in annual updates of BMI changed the fatty liver prevalence (32%) at the age of 50–59 years by −8.0%/10.7%, whereas a 1% decrease/increase in annual updates of the cholesterol ratio changed the prevalence (32%) by −1.6%/1.4%. Therefore, the uncertainty in the cholesterol ratio may not significantly affect the model credibility, although the uncertainty in the cholesterol ratio may be larger than that in the BMI.

#### Internal consistency

The estimates of fatty liver, BMI and LDL–C/HDL–C ratio were all consistent with the estimates from the recorded data of the same cohort. This suggests that our model correctly predicted the annual updates of BMI and the cholesterol ratio based on their predictive algorithms. In addition, using the data of the cohort members who were 30‒39 years of age, we simulated the natural history of fatty liver until they reached 70‒79 years of age. The obtained life‒course of fatty liver was consistent with the trends from the recorded data of the study population in the same age groups (e.g., 40‒49, 50‒59 and 60‒69 years of age). We performed time‒shift simulation of the natural history of fatty liver for different age cohorts (e.g., 30–39, 40‒49 and 50‒59 years of age). The obtained natural history of fatty liver overlapped in the same age groups, although the life‒course of fatty liver changed according to age. This result may increase the credibility of our natural history model.

#### Comparison with external data

Lastly, we compared our fatty liver estimates with external data. The projected prevalence of fatty liver was comparable with that observed in the external cohort whose data were not used to construct our model. However, the simulation overestimated the prevalence of fatty liver specifically at 60–79 years of age. This suspected overestimation may have been partly due to the obtained external data not being cohort data but snap–shot data (data observed for people in different age groups in an observation period). The following two factors may have been associated with this overestimation. First, there was the possibility of selection bias in the external population at this age group; i.e., some older people with fatty liver were unable to participate in the health examinations due to illness or death. Second was cohort effects. Our simulated life-course of fatty liver may not be highly consistent with the external data specifically at 60–79 years of age. However, our fatty liver estimates were within the range of those of external data. In addition, the BMI trends from our model were consistent with those from external data [35]. As BMI is the most important predictor for fatty liver, the external consistency of the BMI trends may increase the credibility of our model.

Based on all of the aspects discussed above, we modeled the natural history of fatty liver with confirmed validity. Annual changes in BMI in a closed population played a strong role in the future prevalence of fatty liver in accordance with accepted clinical phenomena. Therefore, sustainable control of BMI for individuals may be an effective intervention to prevent fatty liver in a population. In the next study, we will use the model for decision analysis. For example, we will be able to determine the best BMI reduction target group from the following groups to prevent fatty liver in a population: (1) the entire population, (2) people with fatty liver and (3) obese people. In the future, the outcomes of this model may help public health managers to make better decisions for reducing the prevalence of fatty liver.

## Limitations

One limitation of our study is that our natural history model simulated a closed population without incorporating mortality. Therefore, the prevalence of fatty liver we simulated may have been slightly overestimated at older ages, specifically for those over 60 years of age. However, we consider our conclusion to be valid for the following reasons: First, the number of deaths can be assumed to be proportional to the number of cases of fatty liver. Therefore, the effects of mortality on the impact of LDL-C/HDL-C ratio are smaller than those of BMI. Second, fatty liver may slowly progress to liver–related or complication–related mortality. For example, Nagaya reported that fatty liver diagnosed in a 42–year–old Japanese man progressed to cirrhosis and hepatocellular carcinoma 20 and 27 years after the diagnosis, respectively [47]. Therefore, a discrepancy in the prevalence of fatty liver between an open model and closed model is more likely to be observed in the older ages, specifically in those over 60 years of age. Fatty liver is most prevalent among people in their 40s and 50s. In this age range, the effects of mortality on the impacts of BMI and the cholesterol ratio can be assumed to be smaller. For these reasons, our conclusion that annual changes in BMI more strongly affected the life‒course of fatty liver than those in the cholesterol ratio is valid regardless of an open or closed population. The feasibility of constructing an open model depends on the availability of data on fatty liver-related deaths (e.g., death caused by liver diseases or complication such as cardiovascular disease).

Another limitation of our study is the lack of longitudinal data for the diagnosis of fatty liver. The sample size of the study population was smaller than in general modeling studies. This may affect the credibility of the model due to potential selection bias. However, the study population with an age range of 30–69 years was almost representative of general Japanese men in the same age groups. In addition, missing fatty liver data for participants in their 30s were input and used for simulation. The agreement between the two sets of projected prevalence data using the estimated fatty liver data and the observed fatty liver data were acceptable (See S2 File). Thus, our model simulated the life–course of fatty liver with confirmed internal validity within the range of external data. Future studies will use additional data to increase the credibility of the model.

Lastly, the model will be influenced by the constantly changing social environment and health care systems. This is an inherent challenge in risk factor/disease modeling. Updating the model will be necessary to make it more credible in the future.

## Conclusion

We modeled the natural history of fatty liver in adult Japanese men. The BMI and LDL–C/HDL–C ratio were significant predictors of incident fatty liver. The effects of annual changes in BMI of individuals on the life–course of fatty liver were stronger than those of changes in the LDL–C/HDL–C ratio. Sustainable BMI control for individuals may be the most effective option for reducing the fatty liver prevalence among Japanese men.

## Supporting information

S1 File Questionnaire for participants on lifestyle behaviors. (DOCX)

S2 File Estimation of missing fatty liver data and validity assessment. (DOCX)

S3 File. Dichotomized variables used in regression analyses for annual updates of the presence of fatty liver (DOCX)

S4 File Determination of predictor variables for the presence of fatty liver (FL) and their predictive algorithms. (DOCX)

S5 File The minimal data set collected from the study population. (CSV)

## Abbreviations

NAFLD: non–alcoholic fatty liver disease
FL: fatty liver
BMI: body mass index
LDL–C: low–density lipoprotein cholesterol
HDL–C: high–density lipoprotein cholesterol
SBP: systolic blood pressure
TG: triglycerides
HbA1c: hemoglobin A1c
