# Correction: Modeling the natural history of fatty liver using lifestyle–related risk factors: Effects of body mass index (BMI) on the life–course of fatty liver

**DOI:** 10.1371/journal.pone.0226059

**Published:** 2019-12-02

**Authors:** Mika Aizawa, Seiichi Inagaki, Michiko Moriyama, Kenichiro Asano, Masayuki Kakehashi

The term “life-course” appears incorrectly in the article title as well as in the Abstract, Data and methods, Results, Discussion, and Limitations sections. In all cases, the correct term is “incidence.” The correct title is: Modeling the natural history of fatty liver using lifestyle–related risk factors: Effects of body mass index (BMI) on the incidence of fatty liver. The correct citation is: Aizawa M, Inagaki S, Moriyama M, Asano K, Kakehashi M (2019) Modeling the natural history of fatty liver using lifestyle–related risk factors: Effects of body mass index (BMI) on the incidence of fatty liver. PLoS ONE 14(10): e0223683. https://doi.org/10.1371/journal.pone.0223683

The term “external cohort” appears incorrectly in the Results and Discussion and in the title of Fig 3. In all cases, the correct term is “external population.” Please see the correct Fig 3 here.

**Figure:**
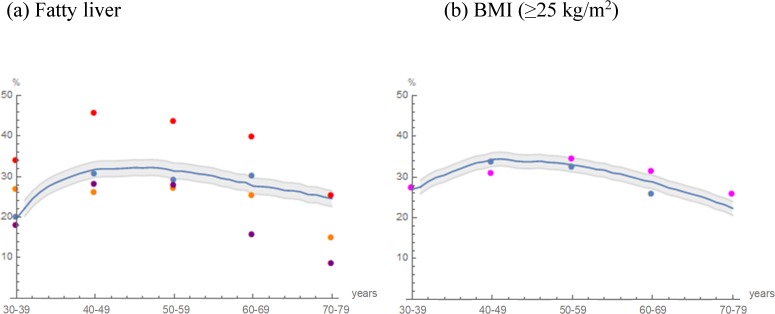
Projected prevalence in cohort 2 and recorded prevalence in external populations: (a) Fatty liver, (b) BMI (≥25 kg/m^2^). **Fig 3** The trajectories of the projected prevalence in cohort 2 (blue curve) are illustrated. Areas in gray indicate the mean prevalence within ±2 SD as Monte Carlo variations. Blue dots indicate the prevalence in the study population recorded in 2012‒2016 (n = 10179). Observed external population data were indicated by purple [29], orange [4], red [28] and magenta dots [35].

The term “Simulated life-course” appears incorrectly in the title of Figs 4 and 6. The correct term is “Projected prevalence.” Please see the correct figures here.

**Figure:**
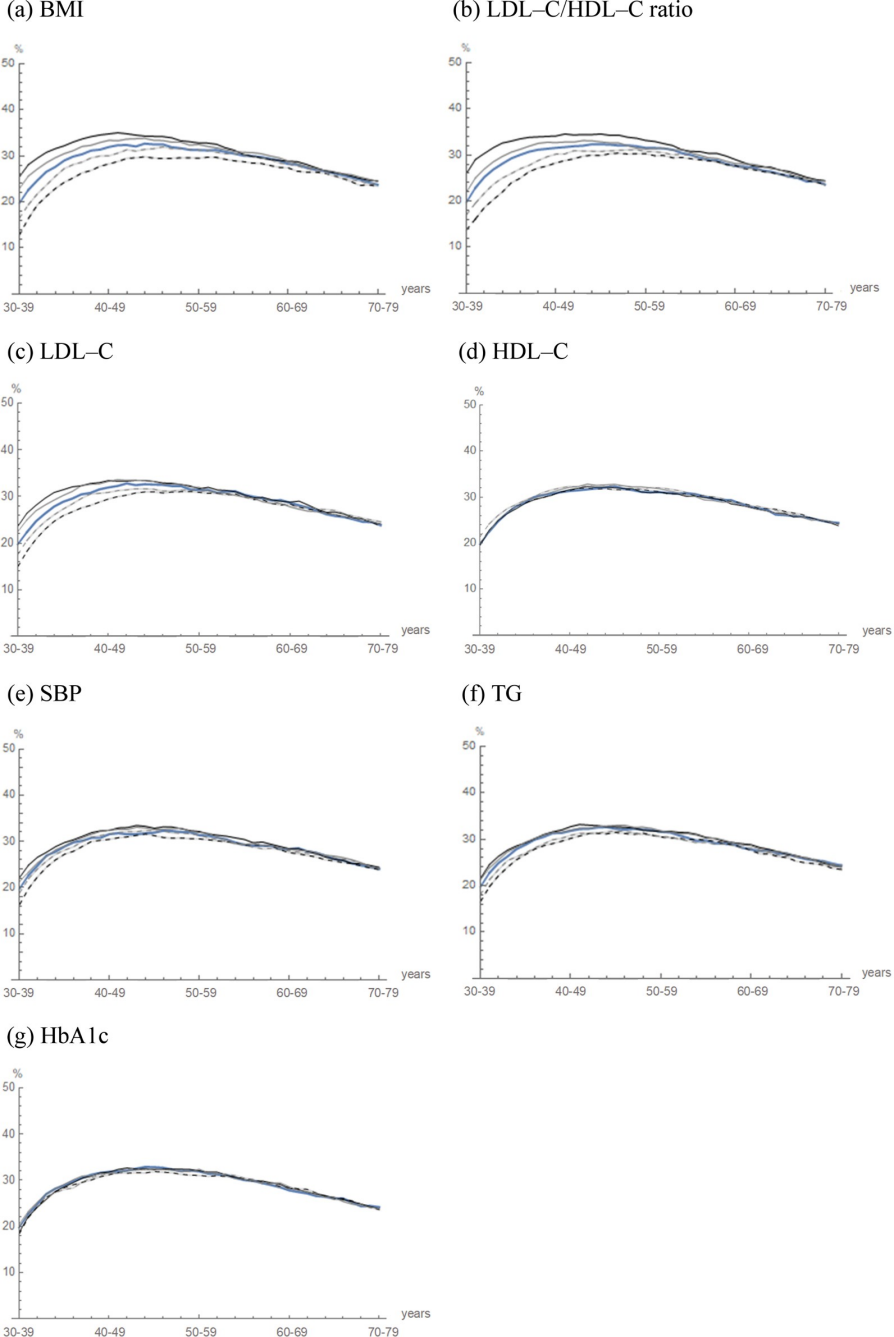
Projected prevalence of fatty liver with variations by changes in the clinical and biochemical predictor variables of cohort 2. **Fig 4** The trajectories of the projected prevalence in cohort 2 (blue curve) are illustrated. The initial proportion of the clinical and biochemical predictor variables of cohort 2 were varied by −40% (dashed black curve), −20% (dashed gray curve), 20% (gray curve) and 40% (black curve).

**Figure:**
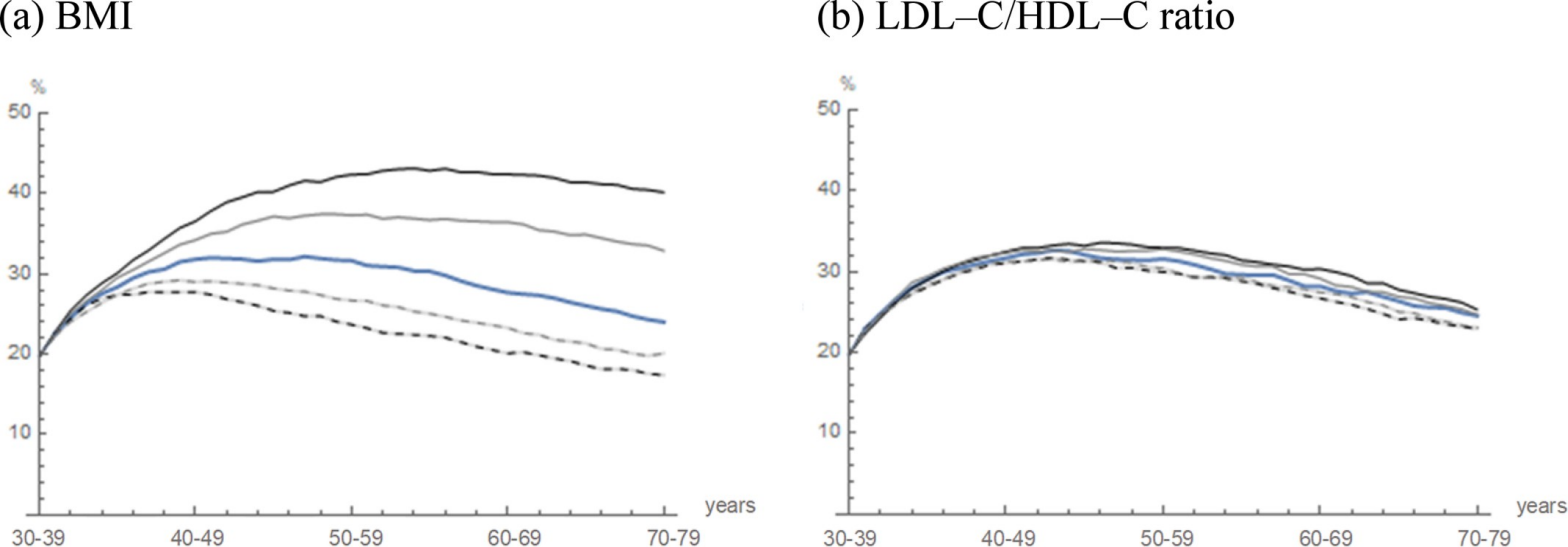
Projected prevalence of fatty liver with variations by changes in the predictive algorithms of BMI and LDL–C/HDL–C ratio. **Fig 6** The trajectories of the projected prevalence in cohort 2 (blue curve) are illustrated. The mean prevalence in which the predicted BMI and LDL–C/HDL–C ratio were varied by −1.0% (a dashed black curve), −0.5% (a dashed gray curve), 0.5% (a solid gray curve) and 1.0% (a solid black curve).

A paragraph is missing from the Simulation and model assessment subsection of the Results. The following paragraph should appear after the first paragraph in the Simulation and model assessment subsection: For cohort 1, the projected prevalence of fatty liver, BMI (≥25 kg/m2) and LDL–C/HDL–C ratio (≥ 2) are shown in Fig 1. Each observed prevalence in cohort 1 fell within ±2 SD of the predicted mean prevalence of fatty liver, BMI (≥25 kg/m2) and LDL–C/HDL–C ratio (≥ 2). This suggested that our model reproduced the observed prevalence of fatty liver by suitably predicting the BMI and cholesterol ratio according to their predictive algorithms.

In the Simulation and model assessment subsection of the Results, there is an error in the sixth sentence of the second paragraph. The correct sentence is: In addition, the fatty liver data of cohort 2 included estimated fatty liver data.

In the Assessment of model performance subsection of the Discussion, there is an error in the fourth sentence of the first paragraph. The correct sentence is: This model captured an increase in BMI up to middle-age, followed by a slow decrease in older age.

## References

[1] Aizawa M, Inagaki S, Moriyama M, Asano K, Kakehashi M (2019) Modeling the natural history of fatty liver using lifestyle–related risk factors: Effects of body mass index (BMI) on the life–course of fatty liver. PLoS ONE 14(10): e0223683 10.1371/journal.pone.0223683 31634357 PMC6802837

