# Supplementary material for: Modeling the natural history of fatty liver using lifestyle–related risk factors: Effects of body mass index (BMI) on the life–course of fatty liver
Source: PLoS One. 2019 Oct 21;14(10):e0223683. doi: 10.1371/journal.pone.0223683 (PMC6802837; doi:10.1371/journal.pone.0223683)
Supplement: S1 File — (DOCX) [file pone.0223683.s001.docx]

**S1 File**

**Questionnaire for participants on lifestyle behaviors**

| **Question** | | **Answer** |
| --- | --- | --- |
| 1 | Are you a smoker?  “Smoker” refers to an individual who has been smoking for ≥6 months or consuming ≥100 cigarettes for at least one month. | Yes No |
| 2 | Have you been exercising at a moderate intensity, i.e. at least 30 min continuously or twice a week for at least one year? | Yes No |
| 3 | How often do you drink alcohol?  (sake, shochu, beer, wine, whisky, brandy, etc.) | everyday |
|  |  | 5–6 days/a week |
|  |  | 4–5 days/a week |
|  |  | 3–4 days/ a week |
|  |  | 1–2 days/ a week  or less |
| 4 | How much ‘sake’ do you drink per day?  Sake (180 ml) is equivalent to the following:  midsize beer (500 ml), shochu (80 ml), whisky (60 ml), two glasses of wine (240 ml) | less than 180 ml |
|  |  | 180–less than 360 ml |
|  |  | 360–less than 540 ml |
|  |  | 540 ml or over |
