# Supplementary material for: Modeling the natural history of fatty liver using lifestyle–related risk factors: Effects of body mass index (BMI) on the life–course of fatty liver
Source: PLoS One. 2019 Oct 21;14(10):e0223683. doi: 10.1371/journal.pone.0223683 (PMC6802837; doi:10.1371/journal.pone.0223683)
Supplement: S2 File — (DOCX) [file pone.0223683.s002.docx]

**S2 File**

**Estimation of missing fatty liver data and validity assessment**

Missing fatty liver data for the 30‒39‒year‒old cohort of the study population (n=1981) were estimated using a criterion obtained by cross-sectional multivariate logistic regression analysis. The determination and assessment of the criterion are detailed below.

**Methods**

**Data and population setting**

The source data for analysis were the data from the study population observed from 2012 to 2016 (presented in the main thesis), which excluded participants missing fatty liver data. Two types of datasets were used. One consisted of data from participants who were 30‒69 years old (n=5115). This dataset was used to derive the criterion for estimation of missing fatty liver data. The other dataset consisted of data from participants who were 30-39 years old (n=181). This dataset was used to estimate fatty liver data using the criterion obtained from the first data set and assess the discrimination ability of the criterion by comparing the estimated fatty liver data with the recorded data.

**Missing value estimation**

***Derivation of the criterion for estimation of fatty liver data***

We conducted cross-sectional multivariate logistic regressions to obtain the most suitable regression equation using the backward elimination method. The obtained equation was used as a criterion to estimate the presence of fatty liver in individuals. In the regression, the following predictor variables were used: clinical and biochemical characteristics (diagnosis of fatty liver, SBP, BMI, TG, LDL-C, HDL-C and HbA1c), and lifestyle (shift work, exercise, smoking and alcohol drinking). The presence of fatty liver was estimated individually using the following two steps: First, we calculated an estimated value for fatty liver using the criterion. Second, we individually predicted the presence of fatty liver when the estimated value was larger than the cut-off value. For the cut-off values, 0.3, 0.4 and 0.5 were applied to assess which value provided the closest prevalence of fatty liver to that recorded. The sensitivity and specificity of the differentiation were calculated by applying each of the cut-off values. The C-index was calculated to assess predictive ability.

***Validity assessment of estimated fatty liver data***

We performed validity assessment by comparing two sets of projected fatty liver prevalence data calculated from two sets of fatty liver data: the estimated set and the recorded set (n=181). To assess agreement between the two sets of projected prevalence, we performed correlation and Bland-Altman analyses.

**Statistical analysis**

All statistical analyses were performed using the software “EZR 1.36 for Windows”. Multivariate logistic regressions were conducted using backward elimination with Akaike Information Criterion (AIC). Bland-Altman plot analysis was performed using the “BlandAltmanLeh” package as an extended function of the EZR system. Pearson product moment correlation tests were performed to evaluate correlations between two groups. A two-tailed P-value p<0.05 was considered significant.

**Results**

**Derivation of the criterion for estimation of fatty liver data**

Cross-sectional multivariate logistic regression revealed the predictor variables and the coefficients, as shown in Table S1. No multicollinearity was found in the regression. The discrimination ability of the regression was quantified by the calculated C-index of 0.86 (95% CI: 0.84 to 0.87).

**Table S1 Predictor variables and coefficients provided by regression to estimate the presence of fatty liver**

| Predictor variables | Coefficients | P-value |
| --- | --- | --- |
| Intercept | −14.8982 | < 0.001 |
| BMI (kg/m^2^) | 0.3280 | < 0.001 |
| LDL-C (mg/dl) | 0.0095 | < 0.001 |
| HDL-C (mg/dl) | −0.0267 | < 0.001 |
| SBP (mmHg) | 0.0103 | < 0.001 |
| TG (mg/dl) | 0.0033 | < 0.001 |
| HbA1c (%) | 0.8559 | < 0.001 |
| Alcohol drinking (yes) | −0.3703 | < 0.001 |
| Smoking (yes) | −0.2085 | 0.01 |
| Regular exercise (yes) | −0.3338 | < 0.001 |
| Shift work (yes) | −0.3028 | < 0.001 |

When the cut-off value was 0.4, the estimated prevalence of fatty liver was 28.1%; this prevalence came closer to that recorded (28.9%) than those obtained when using the cut-off values of 0.3 and 0.5 (See Table S2).

**Table S2 Prevalence, sensitivity and specificity of the model to estimate the prevalence of fatty liver**

| Cut-off value | The estimated prevalence of fatty liver (%) | Sensitivity (%) | Specificity (%) |
| --- | --- | --- | --- |
| 0.3 | 36.7 | 75.4 | 79.1 |
| 0.4 | 28.1 | 64.5 | 86.8 |
| 0.5 | 20.9 | 51.6 | 91.5 |

The estimation was carried out for participants who were 30-39 years old (n=181).

**Validity assessment**

The prevalence of fatty liver for those who were 30-39 years (n=181) was projected based on the estimated fatty liver data (prevalence (E)). This prevalence was compared with that projected using the recorded data (prevalence (R)). Both trajectories of the prevalence demonstrated a good fit, as illustrated in Fig S1.


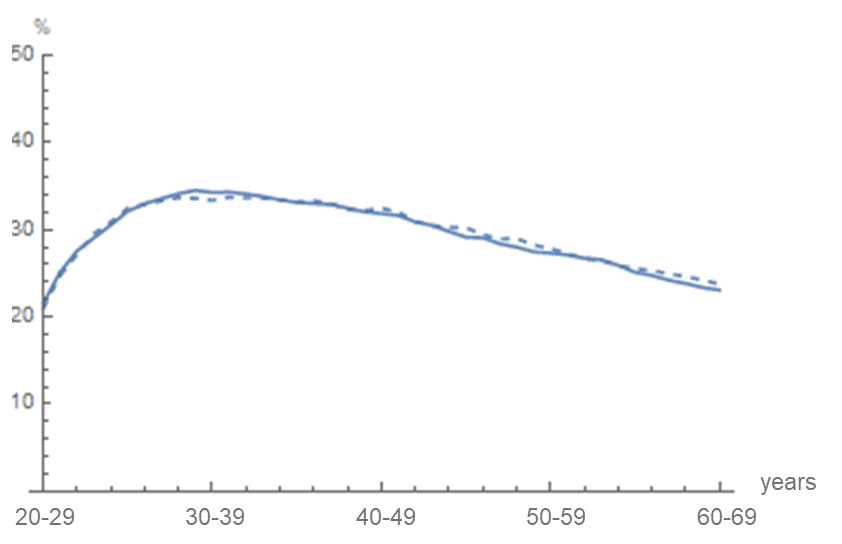


**Fig S1 Comparison of the trajectories of prevalence (E) and prevalence (R)**

The trajectories of the projected prevalence obtained using the recorded fatty liver data (prevalence (R): solid curve) and that obtained using the estimated data (prevalence (E): dashed curve) are illustrated.

Regarding agreement between the two sets of prevalence, prevalence (E) was highly correlated with prevalence (R) (r=0.994, 95% CI: 0.989‒0.997, p<0.001). The Bland-Altman plots further suggested no relationship between the discrepancy and the mean because there was no significant correlation between them (p=0.64). Therefore, the limits of agreement are valid, and the discrepancy between prevalence (E) and prevalence (R) is less than 1% for 95% of individuals. (Fig S2).

**
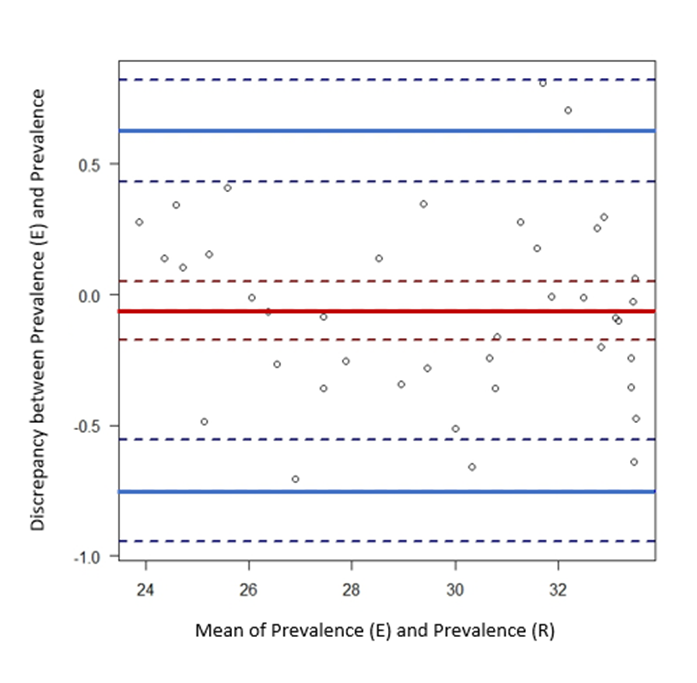
**

**Fig S2 Bland- Altman plots of prevalence (E) and prevalence (R)**

The horizontal axis indicates the means of prevalence (E) and prevalence (R). The vertical axis indicates the discrepancies between prevalence (E) and prevalence (R).

Mean discrepancy: −0.06 (solid red line), standard deviation: 0.35, limits of agreement: −0.75, 0.63 (solid blue lines), mean discrepancy CI: −0.17 to 0.05 (dashed red lines), lower limit of agreement CI: −0.95 to −0.56 (dashed blue lines), and upper limit of agreement CI: 0.43 to 0.83 (dashed blue lines)

**Conclusions**

The criterion to predict missing fatty liver data was determined by cross-sectional multivariate logistic regression analysis. The discrimination ability of the criterion was quantified by the C-index of 0.86 (95% CI: 0.84 to 0.87) between the presence and absence of fatty liver. Moreover, we obtained the correlation (r=0.994, 95% CI: 0.989‒0.997, p<0.001) and the limits of agreement (the lower limit −0.75% and the upper limit 0.63%) between the prevalence of fatty liver projected using the estimated fatty liver data and the observed fatty liver data. This criterion is suitable to estimate the missing fatty liver data for the 30‒39‒year‒old cohort of the study population (n=1981).
