# Supplementary material for: Modeling the natural history of fatty liver using lifestyle–related risk factors: Effects of body mass index (BMI) on the life–course of fatty liver
Source: PLoS One. 2019 Oct 21;14(10):e0223683. doi: 10.1371/journal.pone.0223683 (PMC6802837; doi:10.1371/journal.pone.0223683)
Supplement: S3 File. Dichotomized variables used in regression analyses for annual updates of the presence of fatty liver — (DOCX) [file pone.0223683.s003.docx]

| **S3 File**  **Dichotomized variables used in regression analyses for annual updates of the presence of fatty liver** |
| --- |

| **Variable [Abbreviation]** | **Category** | **Definition** | **Reference** |
| --- | --- | --- | --- |
| Alcohol drinking  **[**ALC**]** (g/day) | Non-alcoholic  Alcoholic | < 30.0  ≥ 30.0 | [1] |
| Body mass index  **[**BMI**]** (kg/m^2^) | Underweight or normal  Overweight or obese | < 25.0  ≥25.0 | [2] |
| LDL-C  **[**LDL**]** (mg/dl) | Recommended  High | < 120  ≥120 | [3] |
| HDL-C  **[**HDL40**]** (mg/dl) | Low  Recommended | < 40  ≥40 | [3] |
| HDL-C  **[**HDL60**]** (mg/dl) | Low or recommended  Recommended | < 60  ≥60 | [3] |
| LDL-C/HDL-C ratio **[**LDL/HDL**]** | Recommended  High | < 2.0  ≥ 2.0 | [4] |
| Triglycerides  **[**TG**]** (mg/dl) | Normal  High | < 150  ≥ 150 | [3] |
| Systolic blood pressure  **[**SBP**]** (mmHg) | Optimal or normal  Hypertensive | < 130  ≥ 130 | [5] |
| Hemoglobin A1c **[**HbA1c**]** (%) | Normal  Suspected diabetes | < 6.5  ≥ 6.5 | [6] |

**References**

1. Watanabe S, Hashimoto E, Ikejima K, Uto H, Ono M, Sumida Y, et al. Evidence–based clinical practice guidelines for nonalcoholic fatty liver disease/nonalcoholic steatohepatitis. *Hepatol Res*. 2015;45(4):363–77.
2. Takahashi H, Mori M. [Characteristics and significance of criteria for obesity disease in Japan 2011]. *Nihon Rinsho*. 2013;71(2):257–61. (in Japanese)
3. Teramoto T, Sasaki J, Ueshima H, Egusa G, Kinoshita M, Shimamoto K, et al. Diagnostic criteria for dyslipidemia. Executive summary of Japan Atherosclerosis Society (JAS) guideline for diagnosis and prevention of atherosclerotic cardiovascular diseases for Japanese. *J Atheroscler Thromb*. 2007;14(4):155–8.
4. Chen QJ, Lai HM, Chen BD, Li XM, Zhai H, He CH, et al. Appropriate LDL-C-to-HDL-C Ratio Cutoffs for Categorization of Cardiovascular Disease Risk Factors among Uygur Adults in Xinjiang, China. *Int J Environ Res Public Health*. 2016;13(2):235.
5. Ogihara T, Kikuchi K, Matsuoka H, Fujita T, Higaki J, Horiuchi M, et al. The Japanese Society of Hypertension Guidelines for the Management of Hypertension (JSH 2009). *Hypertens Res*. 2009;32(1):3–107.
6. Seino Y, Nanjo K, Tajima N, Kadowaki T, Kashiwagi A, Araki E, et al. Report of the committee on the classification and diagnostic criteria of diabetes mellitus. *J Diabetes Investig*. 2010;1(5):212–28.
