# Supplementary material for: Modeling the natural history of fatty liver using lifestyle–related risk factors: Effects of body mass index (BMI) on the life–course of fatty liver
Source: PLoS One. 2019 Oct 21;14(10):e0223683. doi: 10.1371/journal.pone.0223683 (PMC6802837; doi:10.1371/journal.pone.0223683)
Supplement: S4 File — (DOCX) [file pone.0223683.s004.docx]

**S4 File**

**Determination of predictor variables for the presence of fatty liver (FL) and their predictive algorithms**

**Methods**

The predictive algorithm for FL was determined by prospective regression analyses using the data from the study population specified in the main thesis. The following predictor variables were used to predict FL annually: FL, BMI, LDL-C, HDL-C, LDL-C/HDL-C ratio, HbA1c, TG, SBP, age, smoking, alcohol drinking, exercise and shift work. The definitions of the variables are stated in the Methods section in the main thesis. Continuous variables were categorized, as shown in Appendix S3.

Below is the explanation of underlying relationships between input values and outcomes of the model. The conceptual structure of the model is visualized in Fig S1.

**Fig S1 Predictor/predicted variables used for modelling the natural history of fatty　liver using lifestyle-related risk factors**

Predictive algorithms of $V_{j}$(t + 1) were obtained using the Monte Carlo simulation procedure. In logistic regressions, the transition probability was expressed by equations (1) and (2) as an expected value *E* ($V_{j}$(t+1)),

$$E\left( V_{j}\left( t+1 \right) \right)=\frac{1}{1+\exp\left( -{Sc}_{j} \right)} (1)$$

where ${Sc}_{j}$ is

$$\mathrm{Sc}_{j}=\sum_{i} C_{ji}V_{i}\left( t \right)+C_{j0} (2)$$

$C_{ji}$ is a non–standardized regression coefficient and $C_{j0}$ is a constant term which is obtained by multivariate logistic regression analysis. Whether fatty liver is present in the following year, $V_{j}(t+1)$ = 0 or 1 is given by stochastic operation by performing a Bernoulli trial with the probability of the expected value *E* ($V_{j}$(t + 1)).

When *V* is a continuous variable (e.g., BMI), the predictive algorithm of $V_{j}$(t+1) is expressed by the following equation derived from multivariate linear regression.

$$V_{j}\left( t+1 \right)=\sum_{i}^{n} C_{ji}V_{i}\left( t \right)+\delta_{j}, for all j,i=1\sim n (3)$$

Here, *δ* is a constant term that corresponds to a random integer X (X ~ N (μ, σ^2^)) according to normal distributions of regression residuals.

The predictive algorithm for FL was evaluated by the C-statistic and Bayesian Information Criterion (BIC).

**Results**

**The predictive Algorithm for FL**

The results of the logistic regressions used to provide the predictive algorithm for FL (model #1–11) are shown in Table S1. All regression analyses were conducted using forced entry procedures, except for model #2, which was derived from model #1 using the backward elimination method. No multicollinearity was observed between the predictor variables in models #1–11.

Model #1 demonstrated FL, BMI, HDL60, LDL/HDL and HbA1c to be significant predictor variables. We analyzed the recorded data and found that the trajectory of the prevalence of fatty liver was upward convex depending on age. Therefore, we expected age and age^2 to aid in predicting FL and used them as predictor variables in model #1. Contrary to our expectation, these variables were eliminated when the backward elimination procedure was applied (model #2).

The predictor variables in model #2 excluded LDL but included both HDL and LDL/HDL. When HDL in model #3 was replaced by LDL/HDL, model #4 was obtained. Accordingly, the values for BIC decreased from 2445 (model #3) to 2435 (model #4). Therefore, LDL/HDL had a greater role in the prediction of FL than LDL and HDL alone. Conversely, alcohol drinking, smoking, exercise and shift work were not significant predictors for FL (models #8-11), nor was HbA1c (model #6).

TG and SBP were significant predictor variables in models #5 and #7, respectively. However, the BIC values of models #5 and #7 (2438 and 2436, respectively) were larger than that of model #4 (2435). Additionally, the C-statistic value of model #4 was 0.925. Therefore, we selected model #4 as the most suitable predictive algorithm for FL.

**The predictive algorithms for BMI and LDL/HDL**

As BMI and LDL/HDL are included as predictor variables for FL, the variables need to be updated annually in the model. The algorithms for annual changes in BMI and LDL/HDL were determined by multivariate linear regression analyses using the backward elimination method (see Table S2). The following predictor variables were used in the regression analyses: BMI, LDL/HDL, FL, age and alcohol drinking. The continuous variables BMI and LDL/HDL were used without being dichotomized. No multicollinearity was found between the predictor variables in models #12-15. Regarding LDL/HDL, two types of predictive algorithms were obtained for the age ranges of 30-39 and 40-69 years (models #14 and 15). Such age-stratified predictive algorithms fit better with the recorded data from the study population in all ages than the single predictive algorithm in the age range of 30-69 years. Moreover, the single predictive algorithm included age, which was not a significant predictor variable (model #13).

**Table S1 Non-standardized coefficients of predictor variables for FL and statistical parameters of models #1–11**

|  | #1 |  | #2 |  | #3 |  | #4 |  | #5 |  | #6 |  | #7 |  | #8 |  | #9 |  | #10 |  | #11 |  |
| --- | --- | --- | --- | --- | --- | --- | --- | --- | --- | --- | --- | --- | --- | --- | --- | --- | --- | --- | --- | --- | --- | --- |
| Intercept | −3.210 | *** | −3.674 | *** | −3.341 | *** | −3.445 | *** | −3.468 | *** | −3.475 | *** | −3.537 | *** | −3.424 | *** | −3.512 | *** | −3.429 | *** | −3.498 | *** |
| Health Checkup |  |  |  |  |  |  |  |  |  |  |  |  |  |  |  |  |  |  |  |  |  |  |
| FL | 4.029 | *** | 4.096 | *** | 4.066 | *** | 4.060 | *** | 4.016 | *** | 4.067 | *** | 4.053 | *** | 4.079 | *** | 4.092 | *** | 4.082 | *** | 4.069 | *** |
| BMI | 0.773 | *** | 0.846 | *** | 0.907 | *** | 0.892 | *** | 0.865 | *** | 0.832 | *** | 0.845 | *** | 0.915 | *** | 0.928 | *** | 0.939 | *** | 0.889 | *** |
| LDL | −0.033 |  |  |  |  |  |  |  |  |  |  |  |  |  |  |  |  |  |  |  |  |  |
| HDL 40 | −0.060 |  |  |  |  |  |  |  |  |  |  |  |  |  |  |  |  |  |  |  |  |  |
| HDL 60 | 0.425 | * | 0.495 | ** | 0.796 | *** |  |  |  |  |  |  |  |  |  |  |  |  |  |  |  |  |
| LDL/HDL | 0.736 | *** | 0.761 | *** |  |  | 0.921 | *** | 0.869 | *** | 0.972 | *** | 0.921 | *** | 0.928 | *** | 0.944 | *** | 0.934 | *** | 0.922 | *** |
| TG | 0.201 |  |  |  |  |  |  |  | 0.280 | * |  |  |  |  |  |  |  |  |  |  |  |  |
| HbA1c | 0.690 | * |  |  |  |  |  |  |  |  | 0.497 |  |  |  |  |  |  |  |  |  |  |  |
| SBP | 0.254 |  |  |  |  |  |  |  |  |  |  |  | 0.307 | ** |  |  |  |  |  |  |  |  |
| Age | −0.011 |  |  |  |  |  |  |  |  |  |  |  |  |  |  |  |  |  |  |  |  |  |
| Age^2 | 0.000 |  |  |  |  |  |  |  |  |  |  |  |  |  |  |  |  |  |  |  |  |  |
| Lifestyle |  |  |  |  |  |  |  |  |  |  |  |  |  |  |  |  |  |  |  |  |  |  |
| Alcohol drinking | −0.165 |  |  |  |  |  |  |  |  |  |  |  |  |  | −0.126 |  |  |  |  |  |  |  |
| Smoking | 0.022 |  |  |  |  |  |  |  |  |  |  |  |  |  |  |  | 0.110 |  |  |  |  |  |
| Regular exercise | −0.217 |  |  |  |  |  |  |  |  |  |  |  |  |  |  |  |  |  | −0.182 |  |  |  |
| Shift work | 0.078 |  |  |  |  |  |  |  |  |  |  |  |  |  |  |  |  |  |  |  | 0.226 |  |
| Statistical parameters |  |  |  |  |  |  |  |  |  |  |  |  |  |  |  |  |  |  |  |  |  |  |
| C-statistic | 0.933 |  | 0.928 |  | 0.923 |  | 0.925 |  | 0.928 |  | 0.926 |  | 0.928 |  | 0.927 |  | 0.928 |  | 0.929 |  | 0.926 |  |
| 95% CI (upper) | 0.942 |  | 0.937 |  | 0.932 |  | 0.934 |  | 0.937 |  | 0.936 |  | 0.937 |  | 0.937 |  | 0.937 |  | 0.938 |  | 0.936 |  |
| 95% CI (lower) | 0.923 |  | 0.919 |  | 0.913 |  | 0.916 |  | 0.919 |  | 0.916 |  | 0.919 |  | 0.918 |  | 0.918 |  | 0.920 |  | 0.917 |  |
| Degrees of freedom | 3563 |  | 3563 |  | 4276 |  | 4276 |  | 4276 |  | 3876 |  | 4276 |  | 3956 |  | 4108 |  | 4108 |  | 4276 |  |
| BIC | 2057 |  | 1984 |  | 2445 |  | 2435 |  | 2438 |  | 2197 |  | 2436 |  | 2234 |  | 2320 |  | 2319 |  | 2442 |  |

For the abbreviations and definitions of the continuous variables, refer to Appendix S3

*** p < 0.001, ** p < 0.01 and * p < 0.05

**Table S2 Non‒standardized coefficients of predictor variables for BMI and LDL/HDL, and statistical parameters of models #12‒15**

|  |  | **#12** | | **#13** | | **#14** | | **#15** | |
| --- | --- | --- | --- | --- | --- | --- | --- | --- | --- |
| **Predicted variable** | | BMI | | LDL/HDL | | LDL/HDL | | LDL/HDL | |
| **Age range (year)** | | 30‒69 | | 30‒69 | | 30‒39 | | 40‒69 | |
| **Intercept** | | 1.097 | *** | 0.272 | *** | 0.100 | * | 0.510 | *** |
| **Predictor variables** | |  |  |  |  |  |  |  |  |
|  | **Health Checkup** |  |  |  |  |  |  |  |  |
|  | FL | −0.068 | * |  |  |  |  |  |  |
|  | BMI | 0.968 | *** | 0.004 | ** | 0.008 | *** |  |  |
|  | Age | −0.005 | * | −0.001 |  |  |  | −0.003 | * |
|  | LDL/HDL | −0.036 | * | 0.857 | *** | 0.887 | *** | 0.841 | *** |
|  | **Statistical Parameters** |  |  |  |  |  |  |  |  |
|  | Degree of freedom | 4317 |  | 6782 |  | 2459 |  | 4321 |  |
|  | R-squared | 0.928 |  | 0.745 |  | 0.788 |  | 0.718 |  |
|  | Residuals |  |  |  |  |  |  |  |  |
|  | Mean | 0.041 |  | −0.022 |  | −0.029 |  | −0.016 |  |
|  | Standard deviation | 0.887 |  | 0.411 |  | 0.373 |  | 0.429 |  |

*** p < 0.001, ** p < 0.01 and * p < 0.05

**Conclusion**

The predictive algorithm for FL (model #4 in Table S1) was determined through multivariate logistic regression with the C‒statistic of 0.925 (95% CI: 0.916‒0.934). On the other hand, the predictive algorithms for BMI (model #12 in Table S2) and LDL/HDL (models #14 and 15 in Table S2) were determined through multivariate linear regressions with an R‒squared of 0.928 (model #12), 0.788 (model #14) and 0.718 (model #15).
